# Anxiety and depression in emerging adults: The STAND program as a model of scalable screening and intervention

**DOI:** 10.1038/s41386-025-02174-4

**Published:** 2025-08-29

**Authors:** Kate Wolitzky-Taylor, Alainna Wen, Nelson Freimer, Michelle G. Craske

**Affiliations:** 1 Department of Psychiatry and Biobehavioral Sciences, University of California Los Angeles, Los Angeles, CA USA; 2 Center for Neurobehavioral Genetics, University of California Los Angeles, Los Angeles, CA USA; 3 Department of Psychology, University of California Los Angeles, Los Angeles, CA USA

**Keywords:** Psychology, Human behaviour

## Abstract

Depressive and anxiety disorders are highly and increasingly prevalent among 18 to 25 years olds as individuals emerge into adulthood. If untreated, these conditions have potentially serious consequences for social, academic and occupational functioning and confer risk for various mental health and health conditions, rendering prevention and treatment of paramount importance. After reviewing the evidence for prevalence, chronicity and cost of anxiety and depression in this transitional developmental period, we present evidence for psychotherapeutic and pharmacological interventions that can effectively prevent and treat depression and anxiety. Next, we describe the major barriers to prevention and treatment in young adults that result in significant unmet need. To address this mental health gap, we describe a digitally-supported, stratified stepped care system designed to overcome barriers while also personalizing the prevention and treatment of anxiety and depression, called Screening and Treatment for Anxiety and Depression (STAND). We conclude with challenges in this field of research and future.

## Introduction

Anxiety and depressive disorders are increasing at a staggering rate among young adults, aged 18–25 years old [1–3]. Young adulthood is the age group with the highest incidence rates of anxiety and depressive disorders, estimated at approximately 3% each in the United States [4]. This trend has caused concern among the research and clinical communities as well as alarm in the greater public [5–7]. Emerging adulthood is a period of significant life transition coupled with rapid development of prefrontal cortical areas associated with emotion regulation [8, 9], both of which are implicated in the onset and maintenance of anxiety and depression [10–13]. As such, young adulthood is a particularly critical developmental period to target for prevention and treatment. Although a number of interventions have been shown to be effective for anxiety and depression in this age range, there are major barriers to their implementation and accessibility, leading to major gaps in mental health care. There is a need for systems of care that are (1) scalable, (2) maintain fidelity to evidenced-based practices, and (3) adapt to needs as they change over time. Relatedly, young adults would benefit from systems of care that do more than provide access to mental health care but actively manage acute need for more intensive care, suicide risk, and basic needs.

## Prevalence of anxiety and depression in young adults

Depressive and anxiety disorders are highly prevalent and debilitating. According to the National Epidemiologic Survey [14], lifetime prevalence in the United States is approximately 20% for major depressive disorder and more than 30% for any anxiety disorder in adults. Prevalence rates in the United States have been increasing in the past 20 years, most rapidly among young adults ages 18–25 years old [15, 16]. Such trends have been observed across genders and ethnic and racial backgrounds [15, 17]. Moreover, rates of depression and anxiety escalated with the COVID-19 pandemic, especially among young adults [18, 19]. Sex differences become increasingly apparent throughout adolescence and young adulthood, such that females are at almost twice the risk for anxiety and depression [20–22]. While prevalence rates do not differ across racial and ethnic groups within the USA [4, 23], there exist differences in impairment, chronicity, and treatment access. For instance, compared to White Americans, Black Americans report greater functional impairments, Mexican Americans report greater chronicity, and both groups report lower likelihood of receiving psychotherapeutic treatments [23–25].

Anxiety disorders typically onset in adolescence (peak age 15.5 years) and depressive disorders typically onset before young adulthood (peak age 19.5 years) [26]. Emerging adulthood is a particularly high-risk developmental stage, especially in the context of transitioning to college settings [27]. More than half of the students surveyed at a public American university (N ~ 3000) reported anxiety and depression in their last year of studies [28], and prevalence in college students is higher than same-aged peers who are not in college [29]. Transition to college is associated with numerous stressors, including but not exclusive to leaving the family home for the first time, forming new friendships, and irregular daily patterns, all of which can increase risk for depressive and anxiety disorders [30–32]. Together, these data indicate that emerging adulthood and transitioning to college are particularly high-risk developmental periods.

## Implications of untreated anxiety and depression

Untreated depression and anxiety typically present with a chronic course [33] and are associated with reduced quality of life and functioning in the domains of suicidality, physical health, education and work, and relationships [34, 35]. This extends to decreased academic performance [36, 37]. For example, college students with a diagnosis of depression or anxiety and not receiving treatment had significantly lower grade averages than students without a diagnosis [38]. Moreover, depressive and anxiety disorders are highly comorbid with sleep disturbances and impaired memory and attention, all of which impair academic performance and decrease quality of life [39–42]. Relatedly, depressive and anxiety disorders in adolescents and young adults have been linked to impaired social functioning, including smaller social networks, engagement in fewer social activities, and feelings of loneliness [43, 44].

Major depressive disorder is highly comorbid with suicide, with risk of death by suicide almost 20 times higher than that of individuals without major depressive disorder [45]; notably, suicide rates increased significantly in young adults from 2007-2021 [46]. Both depressive and anxiety disorders are associated with increased risk of physical disorders like cardiovascular diseases, diabetes, and cancer [47–49]. Moreover, depressive and anxiety disorders are strongly associated with risk for substance use disorders [50, 51], which is among the most critical problems facing college students in the US [52]. Lastly, perinatal depression and anxiety in mothers are adversely associated with offspring development [53].

## Role of adversity and stress

Early adversity, such as physical and sexual abuse, parental separation, and maltreatment, is associated with increased risk for anxiety and depression [54]. The neural and cognitive pathways impacted by different dimensions of early adversity (e.g., threat and deprivation) are possible mechanisms for increased vulnerability for both conditions [55]. Adversities associated with world-wide pandemics, conflicts, and environmental disasters may well contribute to peaks in the incidence of anxiety and depression, as occurred with COVID-19, which especially impacted younger age groups [56].

Chronic stressful events also predict the onset of anxiety and depressive disorders [34, 57]. These include social determinants of health such as poverty, unemployment, lack of social support, discrimination, bullying, unsafe neighborhoods, and poor housing [58, 59]. Loneliness is a type of chronic stress that confers risk especially for young adults and especially for men [60]; increased loneliness during COVID-19 lockdowns may have exacerbated the escalation in depression and anxiety.

Although social media can counter aloneness, excessive use of social media might function as another form of chronic stress and may contribute to the increasing prevalence of anxiety and depression. For example, depression and anxiety are proportional to the amount of time spent on social media sites, the frequency of usage, and the number of platforms being used [61, 62]. It is speculated that excessive use of social media can lead to lowered self-esteem through unhealthy comparisons, burnout, preoccupation at the cost of other sources of attention and reinforcement, and enhanced social anxiety due to limited social interactions in real life, all of which may be enhanced by pre-existing anxiety [63]. However, effects of social media use can differ across individuals, and there is some evidence for social media to positively impact depression and anxiety via reinforcement of positive relationships [61]. All of these potential pathways await further empirical validation.

## Prevention of anxiety and depression

Prevention efforts are designed for individuals with subclinical symptoms or those at risk for anxiety and depression [64, 65]. The value of early prevention is clear given the childhood, adolescent, and early adulthood onset of anxiety and depression, their typical chronicity if untreated, the risk they confer for other mental health and health conditions, and impairments in quality of life (see section on Prevalence of Anxiety and Depression in Young Adults). Stress generation models, in which depression (and anxiety) contribute to ongoing stressors that in turn magnify depression, provide even more impetus for prevention [66]. The majority of prevention studies target children and adolescents rather than emerging adults, and are primarily comprised of cognitive behavioral therapy (CBT [67]). The effect sizes for the reduced incidence of anxiety and depressive disorders tend to be small, with larger effects for youth at risk for anxiety or depression (e.g., inhibited temperament, depressed or anxious parents) or with minimal symptoms, compared to universal prevention programs [68, 69].

Despite the evidence for effectiveness, prevention programs are rarely implemented, perhaps because they are costly, not easily accessible, and possibly stigmatizing, particularly when delivered in schools [70]. Moreover, persons at the highest risk are often the least motivated to participate in prevention programs [71]. Online or digital therapy prevention programs address engagement issues to some degree, and have been shown to reduce depressive symptoms, including several studies with college students [72]. However, as with in-person programs, effect sizes for digital prevention programs remain small [72].

Prevention strategies that target specific mechanisms are expected to produce stronger effects. One such targeted mechanism that has been evaluated in young adults and college students is repetitive negative thinking, which is a risk factor for poor mental health in general and in college student samples [73] in whom it may be increasing in prevalence [74]. A version of CBT that specifically targets repetitive negative thinking, delivered to high school and university students with elevated repetitive negative thinking, in either group format or internet version, was found to decrease depression and anxiety symptoms and disorder onset over the next 12 months compared to a wait-list control [75]. The effects from the internet version were also larger than effects from usual care in university students [76].

## Treatment of anxiety and depression

Psychological and pharmacological treatments for anxiety and depression have demonstrated robust effectiveness for several decades, although studies rarely target only young adult populations. First line treatments for adolescents and adults include (1) psychotherapies that utilize cognitive and behavioral principles of change, such as behavioral activation, exposure, problem solving, and cognitive restructuring, or interpersonal therapy which focuses upon interpersonal disputes, bereavements, role transitions, and skills; (2) selective serotonin reuptake inhibitors (SSRIs); and (3) serotonin-norepinephrine reuptake inhibitors (SNRIs) (although the latter are not FDA approved for youth under 18). Other psychological and behavioral treatments, including mindfulness-based stress reduction and prescriptive exercise, have consistently demonstrated efficacy in reducing symptoms of depression [77, 78]. Moreover, transcranial magnetic stimulation (TMS) has demonstrated treatment effectiveness for depressive and anxiety disorders in adults [79, 80]. Below we describe some of these interventions and the efficacy data supporting them, drawn mostly from systematic reviews and meta-analyses.

### Psychotherapy

CBT is unequivocally the psychological treatment of choice for anxiety disorders across the lifespan, with moderate to large effect sizes for panic disorder, social anxiety disorder, and generalized anxiety disorder that are maintained over long-term follow-up periods in adults [81, 82]. CBT for anxiety outperforms other psychological treatments such as psychodynamic therapy, relaxation, and supportive therapy [83, 84]. Anxious adolescents and young adults may benefit to a greater degree from CBT than do older adults [85]. Aside from CBT, a limited number of studies demonstrate support for short-term psychodynamic therapy [86] and mindfulness approaches [87] for adult anxiety disorders, without specific evidence for young adults.

CBT for depression is an umbrella term for cognitive therapy (CT) and behavioral activation (BA) therapy, sometimes administered independently. CBT for depression has robust empirical support, with meta-analyses showing moderate to large effects, including in young adults [81, 88–90]. Hence, CBT is recommended as a first-line treatment for depression in adolescents and young adults [91–93]. Aside from CBT, interpersonal therapy [94, 95], problem solving therapy [96], and mindfulness-based stress reduction [78, 97] have all demonstrated moderate to large effect sizes compared to no-treatment or non-CBT control conditions, including among adolescent and young adult populations.

Advanced understanding of mental experiences that are influential for engaging in and benefiting from CBT for anxiety and depression develop during adolescence [98]. Although adolescents and young adults are more mature in their cognitive and social abilities than children, developmentally appropriate considerations are still recommended. Some adaptation examples include focusing upon interpersonal skills, providing more structure and feedback, and for adolescents, involving parents as co-therapists [99, 100].

### Pharmacotherapy

Pharmacotherapy is a more common treatment approach for anxiety and depression, partly because it is frequently offered in primary care rather than specialty settings, even though patients typically prefer psychological treatments, especially younger and female patients [101]. Notably, most pharmacological studies are conducted in either adolescent or adult populations, and very few focus specifically on young adults. Numerous meta-analyses converge to indicate the efficacy of serotonin reuptake inhibitors (SSRIs) and serotonin-norepinephrine uptake inhibitors (SNRIs) for youth and adult generalized anxiety disorder, social anxiety disorder, and panic disorder [102–106], with small to medium effect sizes compared to pill placebo [35]. Few studies directly compare pharmacotherapy to CBT for anxiety disorders. One meta-analysis suggested that pre- to post- effect sizes are larger for pharmacotherapies than CBT [102]: however, comparisons were confounded by sample differences, with greater pre-treatment anxiety severity within pharmacotherapy samples allowing for greater change by post-treatment.

Moderate effect sizes are observed for SSRIs and SNRIs for adult depression compared to placebo controls [107]. As with anxiety disorders, very few studies specifically target young adults, and only SSRIs are FDA approved for depression in youth under 18. Individual patient characteristics have been investigated as predictors of differential treatment response, but with little success to date [108]. There is some evidence that adding adjunctive low doses of second generation antipsychotic medication (e.g., risperidone, aripiprazole) for SSRI/SNRI nonresponders can augment outcomes [109]. However, there has been little investigation of the safety and efficacy of this approach in young adults. Although equivalent at post-treatment, pharmacotherapy may have lower effect sizes than CBT at follow-up, indicating that CBT in adults may be preferable for long-term depression symptom maintenance [110].

Considerations for young adults include adverse effects of pharmacotherapies, social environment, and brain development. First, while the association between antidepressant use and suicidality is complex, there is some, albeit mixed, evidence suggesting an age-dependent relationship between antidepressants and suicidality. Specifically, some studies have shown that while antidepressants are protective against suicidality in adults aged 30 years or older, they may increase the risk of suicidal ideation (and possibly suicidal behavior) in youth [111, 112]. Young adults are also more likely to have physical adverse effects including reduced growth and reduce bone-mass density [113, 114]. In terms of the social environment, adolescence and young adulthood are critical times of social transition, wherein peer interactions have a stronger influence on wellbeing and functioning [115]. There is evidence that response rates for antidepressants are higher, for instance, for young adults in supportive social environments [116, 117]. Lastly, decreased response to rewards, which has been implicated in depressive disorders, may be more prominent in adolescents and young adults because of changes in the dopamine system and reward function [118]. For instance, adolescents show altered striatal response to reward compared to children and adults, a process that is dopaminergically mediated [119]. This altered reactivity in the striatum is also associated with poorer response to treatment [120]. Therefore, puberty-related changes in reward function might at least partly explain the poor response to SSRI treatment in young people observed in some trials [121]. Overall, there is a scarce literature on pharmacological treatments in adolescents and young adults specifically and the associations discussed here are from a small set of studies. Therefore, there is a need for future research to focus on this area.

### Combined psychotherapy and pharmacotherapy

There is some evidence for benefits from combining pharmacotherapy and psychotherapy for adult depression [110] but not for adolescent depression [122]. The benefit from the combined approach for adult depression does not remain when psychotherapy is limited to CBT [89, 110]. The evidence for combined treatment approaches is mixed with respect to anxiety disorders, with some studies showing benefits in the short-term, including youth and adolescent samples [123, 124], and others not [125, 126]. The combination may be detrimental in the long term [127, 128], possibly due to failure of learning acquired in the context of a drug state to transfer once pharmacotherapy is discontinued [129]. However, we are not aware of long-term detrimental effects from combined approaches for anxious adolescents and young adults.

### Emerging psychological and biological treatments

Various psychological augmentation strategies are being investigated in adults with anxiety disorders, including inhibitory retrieval approaches to exposure therapy [130], reduction of safety behaviors [131], management of intolerance of uncertainty [132], and values-based exposure [133]. A variety of pharmacological strategies are being tested to augment exposure therapy, such as d-cycloserine, L-DOPA, and glucocorticoids [134]. Newer neuro-modulatory methods are being explored including low intensity focused ultrasound pulsation targeting the right amygdala in those with treatment-resistant generalized anxiety disorder [135]. Transcranial direct current stimulation has been shown to improve outcomes when combined with CBT relative to CBT alone for generalized anxiety disorder [136]. To our awareness, none of these emerging treatments have been tested specifically in young adults with anxiety disorders.

Given evidence for low positive affect and reward deficits to characterize depression and anxiety [137], newer psychological treatments specifically aim to improve the anticipation, attainment, and learning of reward (e.g. Positive Affect Treatment, Amplification of Positivity Treatment). They have been shown to be effective for mixed depressed and anxious samples [138–141]. These positive-emotion focused treatments are demonstrating superiority to “standard” CBT [138, 141], and address a major unmet need and preferred patient outcome [137].

Emerging biological treatments for depression include ketamine and intranasal esketamine, which are FDA-approved and are believed to exert effects through glutamatergic modulation [142, 143] and neuroplasticity facilitation [144, 145]. However, long-term effects remain uncertain [146, 147]. There has been interest in psilocybin, typically provided in combination with psychotherapy, the latter of which is often undefined [148]. Newer versions of TMS are accumulating evidence for adult depression, such as accelerated TMS, theta burst stimulation, and low field magnetic stimulation, all still primarily focused upon the dorsolateral prefrontal cortex [148]. However, the extent to which these newer versions are more effective than standard rTMS is unclear [148]. There is evidence for bright light therapy to enhance the effects of standard rTMS [149].

Anti-inflammatory agents, such as non-steroidal anti-inflammatory drugs that are combined with antidepressants, are showing promising effects for depression [150], as are probiotics which are specifically suggested for young adults where they may limit the negative effects of stress on neural development [151]. Aside from probiotics, none of the emerging treatment augmentations or treatment approaches for depression have been specifically designed for or tested in young adult samples.

## Barriers to treatment and need for scalable models of care in young adults

Despite the prevalence and impairment of depressive and anxiety disorders, it is estimated that only half of individuals with depressive disorders and a third with anxiety disorders seek professional help [152–154]. Use of mental health services is especially low during young adulthood, when only approximately one-quarter to one-third of those experiencing depressive or anxiety symptoms seek professional help or receive mental health services [155, 156]. Major barriers to treatment seeking include stigma and embarrassment, concerns about confidentiality and trust, as well as lack of accessibility due to constraints related to time, transportation, and cost of treatment [157, 158]. Other barriers among young adults include lack of knowledge about mental health services, fear of seeking help, and preference for self-reliance [157, 158].

Once treatment is sought, further barriers include low availability of clinicians trained in evidence-based treatments, lack of referrals to appropriate treatments, lengthy waiting times and delays caused by poor coordination among the health care tiers [159]. Moreover, for individuals who receive evidence-based treatments, engagement rates are often low. Attrition from CBT, for example, can range from approximately 25% to 50% in RCTs and is likely to be higher in community settings [160–162]. Attrition for pharmacological treatments can range from approximately 15% to more than 50% (see [163], [164], and [165] for reviews), with higher attrition in younger individuals [164, 166, 167]. Attrition is likely to be driven in part by the same barriers as those impeding initial treatment uptake. Thus, there is a need to address barriers to treatment engagement to improve access to mental health care and treatment outcomes.

## Value of digital mental health

One solution to unmet need is digital mental health care, given its lower economic costs, accessibility at any time and place, ease of access to a wider range of people (disabled population, rural areas, etc.) and the reduction in waiting time [168]. Digital therapies are now established as effective tools for managing depression and anxiety, with most evidence for CBT digital therapies [169], including for college student samples [170, 171]. Digital therapies for depression and anxiety are widely accepted [172], and there is little difference in the effects of digital CBT compared to face-to-face CBT for anxiety and depression [172, 173]. However, there have been no reported comparisons between digital therapy versus face-to-face therapy combined with pharmacotherapy, which is a relevant comparison for more severely depressed samples for whom combined approaches are recommended [162]. Mental health related apps are also gaining empirical support for depression and anxiety [174], although effect sizes are small and few are derived specifically from evidence-based therapies.

There is some evidence for larger effects when digital interventions are provided with support (via coaches, therapists, administrators) relative to unguided interventions, especially synchronous support [175–178] and in particular for more severe depression [179]. Mental health care models that combine non-specialists and digital mental health innovations have unique potential to expand the reach of and engagement with high-quality evidence-based therapies [180]. Non-specialist guidance might come in the form of peer coaches [181] who share communities, identities, or lived experiences, and limit obstacles to care, such as lack of trust, stigma, and cultural and linguistic barriers. In contrast to unstructured peer recovery models sometimes used for substance use disorders [182], peer coaching to support digital therapies for anxiety and depression involves highly structured interactions with extensive training, certification and supervision [180].

## STAND program as a scalable model for screening and intervention

### Overview

In response to the staggering need for increased access to evidence-based prevention and treatment for anxiety and depression among young adults, our research team developed a tiered system of care called Screening and Treatment for Anxiety and Depression (STAND), which we implemented at a four-year university [183] and are currently rolling out at community colleges [184].^1^ The first implementation at a four-year university was delivered within the context of a research protocol. The second implementation consisted of a pilot demonstration project (i.e., non-research) at a community college in partnership with their administrators and a county-level Department of Mental Health. The third implementation of STAND (which is ongoing) is a large-scale effectiveness/implementation hybrid trial at the same community college.

The STAND program was designed to address a multitude of needs through a set of guiding principles. These *principles* include (1) scalability of access through digital technologies, (2) evidence-based care, and (3) personalization through measurement-based care. The *needs* that are addressed include personal and structural barriers to mental health care [157, 158]; these are addressed through the provision of online, digital screening, and wherever possible digital therapy. STAND digital screening extends to suicidality in order to facilitate rapid detection and management of self-harm, which is another critical need in young adults [46]. Given the contributory role of chronic stress and social determinants of mental health [58, 59], the STAND digital screening program additionally assesses and manages basic social needs.

Ongoing, measurement-based care is a hallmark of the STAND program, with weekly measurement of symptoms of depression, anxiety, and suicidality, as well as less frequent but repeated measurement of basic needs. In addition to risk and basic needs management, measurement-based care addresses the needs for personalization in several ways. First, it addresses the need for different levels of care by using a stratified stepped care approach [185, 186], dependent upon presenting symptom severity. Second, it addresses the preference for interventions tailored to personal problem areas compared to generic CBT, by measurement-based selection of CBT modules [188]. Third, it addresses changing needs over time by guiding efficient adaptation of level of care [189, 190]. Finally, the STAND program addresses the need for prevention, to limit the scarring and stress generating effects of depression (and anxiety) [66], as well as the need for acute treatment. Therapeutic strategies for both the prevention and acute treatment components of STAND are evidence-based.

**Fig. 1:**
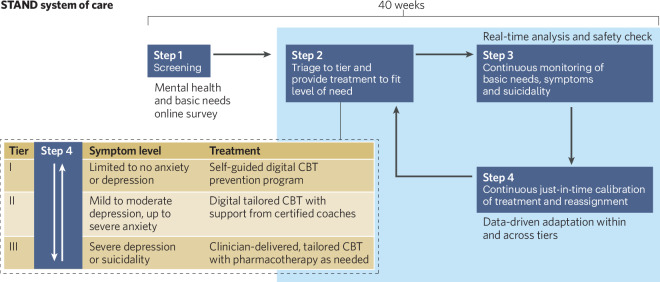
A diagrammatic representation of the STAND system of care.

In summary, STAND is a digitally-supported system that uses computer adaptive testing for anxiety and depressive symptoms to initially triage students to an appropriate level of care, followed by frequent monitoring of symptoms to adapt level of care as needed over a period of 40 weeks (or more). Suicide risk and basic needs monitoring and management are integrated into the digitally-supported assessment system. The levels of care range from (1) a self-directed digital CBT prevention program targeting repetitive negative thinking, for those with minimal to no symptoms of anxiety or depression, to (2) personalized and modularized digital CBT supported by trained and supervised peer coaches (typically other students), for those with moderate depression or moderate to severe anxiety, to (3) face-to-face individual CBT with the option for medication management (with an exception for bipolar disorder), delivered via telehealth, for those with severe depression symptoms or at risk of suicide. Thus, unlike a myriad of other digital therapies, the STAND program is not limited to digital therapy. Clinical care is included because, even though severity of depression has not been shown to moderate the effects from digital CBT vs. face-to-face CBT (e.g.,[191]), there is no evidence to date to suggest that digital CBT alone is as effective as face-to-face CBT combined with medication for severely depressed and actively suicidal samples. It is most likely for this reason that treatment guidelines, such as NICE [192], recommend digital therapies (and other lower intensity treatments) for mild to moderate depression and higher intensity clinical care for more severe depression. See Fig. 1 for a diagrammatic representation of the STAND system of care.

### Assessment, monitoring, triaging, and treatment adaptation

Within STAND, symptoms are measured at baseline and weekly thereafter (for the period of study enrollment) using an online tool, the Computerized Adaptive Test for Mental Health (CAT-MH; see [186, 193] for review). The CAT-MH is a suite of adaptive mental health tests (e.g., anxiety, depression, and suicide severity) based on multidimensional item response theory. The CAT-MH has been shown to increase measurement precision and reduce burden relative to traditional fixed length instruments. It is suitable for large scale deployment and is optimal for weekly assessment because it is briefer than standard questionnaire batteries (< 5 min) and because the adaptive nature minimizes responder bias that evolves with excessive repetition of the same item set. Reliability and validity of the CAT-MH scales for depression, anxiety, and suicidality have been demonstrated [194–196]. Access to the CAT-MH is obtained through a paid license from Adaptive Testing Technologies, based in Chicago, IL. It can be implemented via the vendor’s user portal or API integration. The assessments are available in English and Spanish.

The CAT-MH digital tool is the primary driver of data-driven assessment and monitoring to (1) initially triage individuals to a level of care and (2) continuously monitor symptoms to adapt level of care over time. Data-driven triaging to a level of care may improve upon standard stepped care models, in which everyone begins with the lowest level of care, by personalizing treatment selection rather than waiting for failure to respond to a lower level of care before stepping up to a higher level [186]. Standard stepped care can lead to symptom worsening and dropout [197, 198], whereas a stratified approach may reduce dropout and improve time until symptom remission [187]. As described, those with no to mild symptoms of depression or anxiety are triaged to self-guided digital prevention, those with moderate depression or up to severe anxiety are triaged to coach-guided and tailored digital CBT, and those with severe depression or suicide risk are triaged to clinical care.

Throughout active treatment, those initially triaged to self-guided digital CBT prevention or coach-guided digital CBT are switched to clinical care if their CAT-MH scores elevate to indicate severe depression or risk of suicide. Conversely, severely depressed students assigned to clinical care are moved into digital CBT with coaching as their depression lessens to moderate or lower levels on CAT-MH assessments, thus reserving clinicians for the more severe, acute patients while maintaining gains via digital therapies. Following treatment, participants are encouraged to continue weekly CAT-MH assessments in case re-initiation of care is indicated. Specifically, scores that increase ≥ 30% from scores at treatment completion trigger outreach to re-engage in care if that score exceeds the mild range. This adaptation approach is in line with the concept of clinical staging or recognition that needs change over time due to a myriad of factors (e.g., waxing and waning nature of the disorder, moderating influences such as ongoing life stress, and response to interventions) [187, 193, 199]. It also improves upon the standard practice of waiting until a patient has not responded to a full course of treatment before adjusting/personalizing to optimize care.

Another key feature of STAND is ongoing suicide risk monitoring and response, of particular importance given the high rates of suicidality in young adults [200] and college students [201] and low rates of help-seeking [202]. In STAND, further assessment is conducted at every endorsement of suicidality on CAT-MH scales throughout program enrollment: endorsement of suicidal ideation with either a plan or intent in the past month, or active preparations to take their own life in the past three months, results in a computer-automated alert to a team who conducts outreach and safety assessment, with reports back to administrators and clinical staff, who then follow-up as needed. A positive suicide risk assessment activates initial triaging to or moving up to clinical care. Of note, lower levels of suicidality (e.g., ideation with no plan or intent) are included in the CAT-MH suicide risk assessment and continue to be assessed and monitored, but they do not reach the threshold for imminent risk concern indicating that outreach is needed.

Given the role of social determinants in mental health [58, 59], STAND includes assessment and management of basic needs including food, housing, and other resources. In California community colleges, 50% of students reported food insecurity in the prior 30 days, 60% were housing insecure, and 19% were homeless [203]. Not surprisingly, these stressors negatively impact students’ academic performance, physical health, and mental health [204–209]. Moreover, students with depression and anxiety have even higher rates of food and housing insecurity [210]. Students in STAND complete a basic needs assessment at baseline and every eight weeks throughout their participation (as needs may change over time). A licensed social worker outreaches to those whose responses indicate a need for resources, and agree to be contacted, and conducts additional assessment and linkage to basic needs services in the community. The extent to which the addition of management of basic needs improves uptake and efficacy of STAND is still being investigated.

In addition, STAND screens for psychosis and severe substance use. Responses to initial web-based screening tools (i.e., Prodromal Questionnaire – Brief Version [211] for psychosis, and an internally-developed assessment for severe substance use patterns such as daily cannabis use, frequent use of stimulants and opioids, or any use of fentanyl) trigger alerts to a licensed clinical social worker who contacts the participant for further assessment and possible referral to specialty care. Because psychotic and severe substance use disorders are outside of the scope of STAND, individuals identified with these problems are excluded from participation in STAND.

### STAND tiers of care: prevention CBT, digital CBT with coaching, and clinical care

The lowest tier is a digital CBT prevention program, which is assigned to those with minimal to no symptoms as a self-directed web-based prevention tool. This prevention program is specifically targeted at negative repetitive thinking, adapted from rumination-focused CBT which has demonstrated efficacy for depression and anxiety in college samples [75, 76]. It contains modules such as problem solving, concrete thinking, relaxation, mindfulness, self compassion, and improving relationships through interpersonal effectiveness/assertiveness skills.

The middle tier is tailored digital CBT with coaching, which is assigned to those with moderate depressive symptoms or up to severe anxiety symptoms, and consists of online CBT modules developed by the UCLA Depression Grand Challenge (DGC) [180], supported by coaches through video chats. All digital modules are evidence-based and address depression, anxiety and worry, panic, social anxiety, trauma, and sleep dysregulation. They include behavioral activation, self-monitoring, exposure therapy, cognitive restructuring, self compassion, relaxation, mindfulness, elements of positive affect treatment, and sleep regulation. The latest advances such as the inhibitory retrieval approach to exposure (derived from extinction learning principles rather than fear habituation) [121] and positive affect treatment for increasing reward processing (comprised of strategies to increase anticipation and savoring of rewarding experiences, attention to and memory for rewarding experiences, and learning which actions lead to more rewarding outcomes) [137] are incorporated. An embedded adaptive measurement-based system directs students to the digital content most relevant for their symptoms. Each 30 to 40-minute online lesson teaches skills through text, graphics, audio, video, and quiz content, designed to facilitate retention. Homework exercises are supported by an app toolbox accessible on smartphones and mobile devices as well as additional worksheets that can be downloaded.

Peer coaching is included to increase engagement and outcomes, as has been shown in other research [175]. Moreover, coaches can decrease loneliness, which is a known risk factor for anxiety and depression [60]. Coaches (themselves college students) provide up to eight 30 min one-on-one video sessions to their assigned participants. Coaches use process skills and motivational interviewing to increase engagement, support and encourage the application of CBT skills, and problem solve barriers to skill utilization. The coaches are trained and certified by licensed clinicians in foundational CBT skills, active listening, empathic responding, motivational support, confidentiality, and ethical decision making. Licensed clinical supervisors are available any time a coaching session occurs in case a clinician is needed for support.

A detailed description of the coach training process, which has been iteratively modified and improved upon over time, can be found in [180]. Briefly, in its current format, coach training is a 15-week certification process that combines didactic instruction with experiential learning, including asynchronous coursework (videos, readings, and coach demonstrations) and weekly sessions with coach trainers focused on discussion and role-play practice. Certification is awarded upon successful completion of coursework and skill demonstration via mock coaching sessions, which is evaluated using a behavior-based rubric that assesses foundational coaching skills and the trainee’s ability to apply STAND module content. Experienced coaches have the option to undergo an additional ~15 h of advanced training to coach the exposure-based trauma and panic modules using the same procedures as described above.

Participants with severe depressive symptoms or significant suicidality are initially allocated to clinical care, the highest level of care in the STAND system. This consists of weekly CBT sessions by doctoral students in clinical psychology, psychiatry residents, and masters’ level clinical staff, with the goal of 12–16 sessions but more as needed (i.e., if CAT-MH scores remain in the severe range). CAT-MH scores are monitored weekly by clinicians through the STAND dashboard in order to adjust the therapeutic approach. CBT is modularized (paralleling the digital CBT) and personalized to the patient’s needs. A functional assessment based on a semi-structured interview developed for STAND that incorporates standardized questions with clinical judgment and is administered at intake to guide selection of a CBT strategy most suited to the principal problem. Examples include behavioral activation for depressed mood/inactivity (with latest advances from reward-based approaches) [137], cognitive restructuring for excessive worry, and distress tolerance skills for affective instability/self-harm/chronic suicidality. If the first-line strategy does not result in meaningful change in CAT-MH scores after six sessions (i.e., scores remain in severe range), clinicians revise the functional assessment and consider shifting to a second-line strategy, after consultation with their licensed supervisor. Interventions to address patient non-adherence, acute suicidality, and major life stressors are incorporated as needed. Given the evidence to support the efficacy of CBT, whether alone or in combination with medications [81–85, 88–90, 212, 213], the potential risks of pharmacotherapies in younger persons [111–114, 121], and younger persons’ preference for psychological treatments [101], medications are not a first-line treatment or a monotherapy. One exception is that patients with bipolar disorder are immediately referred to psychiatry for medication management. Patients who are not responding to CBT alone and remain in the severe range of depressive symptoms after 4-6 sessions are also referred to psychiatry. Medication prescriptions follow best practices for depression treatment, based on the STAR*D algorithm [166].

**Fig. 2:**
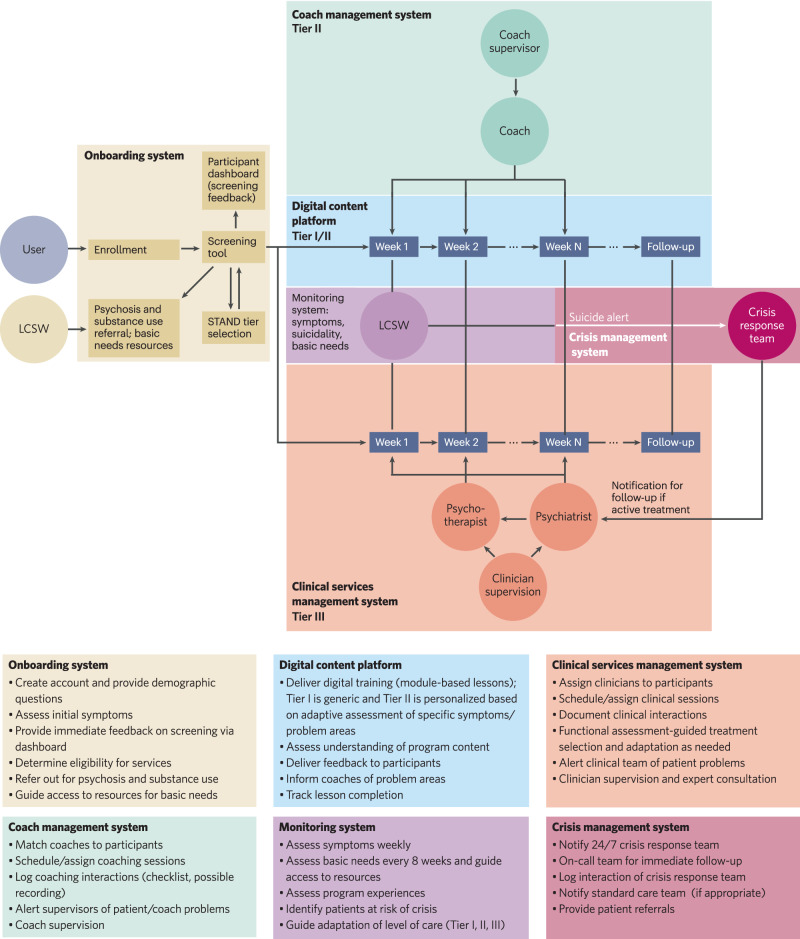
Illustration of STAND systems to manage assessment, risk management, care, and supervision. Modified version of illustration courtesy of Chris Douglas, MD, Department of Psychiatry, Washington University School of Medicine, St Louis, MO, USA.

The entire STAND system is fully integrated through an IT infrastructure to manage the multiple systems of care, oversight and supervision (see Fig. 2). IT infrastructure supports the web-based registration system in which participants undergo initial screening, complete assessments, and are triaged to the appropriate level of care. The IT system collects data directly through STAND assessment systems, routes participants to the CAT-MH for additional assessment, and includes an alert system that communicates with 24/7 crisis teams (a third-party vendor) who manage suicide risk management and with the clinical teams. It also manages the digital platform for Tiers I and II and a clinician dashboard for Tier III, in which clinicians are able to view participant symptom scores and document their clinical services, and uses ongoing data collection to guide the adaptation to either higher or lower level of care as indicated. Not shown here are system-generated reminders to participants to complete assessments, lessons, coaching sessions, or clinical visits.

### Proof of concept trials in young adults

Table 1 summarizes the main findings of the two proof of concept trials, including engagement and outcomes. Further details can be found in [183, 214]. Findings from an open trial of STAND with four-year university students (Fall 2017-Fall 2019, sporadically) demonstrated the reach and impact of the program on clinical outcomes: 4845 students were screened for anxiety, depression and suicidality, 3580 (73.8%) were offered STAND care, of whom 516 (14.4%) initiated STAND care. CAT-MH scores on anxiety and depression declined significantly within each level of care, as did suicidality within the middle and top tiers [183]. Additionally, 327 suicide risk alerts were detected and addressed at screening, as were 1054 risk alerts (indicating risk of suicide or severely worsening depression) during the course of the study. No suicides were recorded during the course of the study. Two known emergency room visits and one known hospitalization due to mental health conditions occurred during the course of the study. The program was viewed as acceptable. Specifically, participants (across tiers) rated their care as highly logical (mean scores by tier ranging from 6.65 to 7.60 out of 9), that the program succeeded in meeting their expectations (mean scores by tier ranging from 3.47 to 3.95 out of 5), that they were satisfied with the program (mean scores by tier ranging from 5.24 to 5.90 out of 7), and that they would recommend it to a friend (mean scores by tier ranging from 5.47 to 6.14 out of 7).

**Table 1:** Summary of Findings from the UCLA Trial and from STAND at ELAC.

Variable	UCLA Trial (*n* = 516)	ELAC Evaluation (*n* = 327)
*Tier 1 (lowest tier)*		
Number of participants	180	16
Mean # digital lessons (SD)*	4.22 (1.81)	1.69 (0.87)
Mean # coaching sessions (SD)	0.88 (2.07)	N/A
*p*-value for anxiety outcomes	<0.001	0.059
*p-*value for depression outcomes	<0.001	*Ns*
*Tier 2 (middle tier)*		
Number of participants	197	207
Mean # digital lessons (SD)	4.09 (2.06)	2.73 (2.03)
Mean # coaching sessions (SD)	2.09 (2.56)	2.58 (2.12)
*p-*value for anxiety outcomes	<0.001	<0.0001
*p-*value for depression outcomes	<0.001	<0.0001
*p-*value for suicide risk outcome	<0.001	<0.001
*Tier 3 (highest tier)*		
Number of participants	139	104
Mean # clinical visits	13.86 (7.94)	16.35 (11.71)
*p*-value for anxiety outcome	<0.001	0.013
*p*-value for depression outcomes	<0.001	<0.0001
*p-value* for suicide risk	<0.001	<0.00001

The lowest tier in the UCLA STAND project involved coaching and the same digital program as the middle tier, whereas refinements for STAND at ELAC resulted in removing coaching from the lowest tier and instead using a briefer, self-guided, online prevention program.

The second implementation of STAND in young adults was a non-randomized evaluation of STAND in a Los Angeles community college in partnership with the Los Angeles County Department of Mental Health (DMH; March 2021–June 2023). Prior to enrolling students, STAND was adapted for a community college serving primarily Latinx students with diverse socio-economic backgrounds. We sought input from the community college’s students, faculty, and administrators as well as the Chancellor’s Office for California Community Colleges and the California Community College Health and Wellness Association. Feedback led to the (1) integration of crisis teams between the STAND team, community college resources, and Los Angeles County DMH resources; (2) addition of a social worker to assess and provide linkage and referral for basic needs, in coordination with community college campus resources; (3) revision of the digital CBT (Tiers I and II) to include diverse characters and examples relevant to the students’ life experiences, with repeated user testing and feedback from student focus groups, as well as inclusive language in all materials; (4) embedding STAND within local campus health center resources; (5) inclusion of college faculty and administrators in the design and implementation of recruitment strategies; and (6) training of community college students to serve as peer coaches for the middle tier of treatment. Coordination across the systems on a regular basis ensured successful implementation.

In this pilot evaluation, 2099 students were screened, and 1423 (67.8%) were offered STAND care, of whom 327 (23.0%) initiated care. CAT-MH scores for anxiety and depression significantly declined within each level of care, as did suicidality scores within the middle and top tiers [214]. In addition, 117 screens for substance use or psychosis, 195 suicide risk alerts, over 200 requests for help with management of basic needs were managed, and 103 coaches were trained. No hospitalizations, emergency room visits, or suicides were recorded during the course of study. As with the initial implementation, participants viewed the STAND program favorably: high overall satisfaction (across tiers) was observed on the Customer Satisfaction Questionniaire-8 [215] (mean= 25.2 out of a possible 32; SD = 5.7).

Taken together, these findings support the utility of STAND in identifying and addressing suicide risk as well as in allocating resources effectively to improve clinical outcomes in primarily young adult college students with anxiety and depression across a range of severity.

Notably, both of these initial pilot implementations, although with large sample sizes, were open trials. Further, although clinicians underwent rigorous training to deliver STAND (coaching and clinical care) and were provided with weekly supervision that included review of recorded sessions, no formal independent fidelity checks were conducted. These limitations were addressed in our current implementation of STAND (described below), which includes a randomized controlled design and multiple checks on treatment fidelity.

### Current and future STAND projects

We are currently investigating ways to improve the effectiveness, implementation, and scalability of STAND at community colleges (*N* = 1000 who initiate STAND care), supported by a NIMH-funded ALACRITY Center (P50MH126337). One method for improving effectiveness is to enhance decision making tools for triaging and adapting level of care, which initially relied solely upon symptom severity. We are now investigating whether outcomes are improved by predictive algorithms using multiple baseline predictors (for triaging) and time varying, dynamic predictors (for adaptation) of outcome. Modeling using multiple static features at baseline (e.g., symptoms, age, employment, disability and functional impairment, comorbidity between depression and anxiety, and treatment expectations) has been shown to improve efficiency of stepped care models [216] and dynamic prediction models are showing promising results [190]. Within STAND, the candidate predictors in our triaging and adaptation algorithms are guided by four distinct but overlapping constructs: (1) social determinants of health (e.g., food and housing insecurity, social support, experiences of discrimination), (2) life adversity and ongoing life stress, (3) comprehensive mental health status (e.g., comorbidity, emotion dysregulation, symptom severity, neurocognitive functioning), and (4) enabling (e.g., social support for treatment seeking), predisposing (e.g., stigma and beliefs about treatment), and need (e.g., perceived need for treatment) factors influencing treatment utilization. The candidate predictors within each construct have been shown to confer risk for anxiety and depression [34, 54, 57–59], influence treatment utilization, or predict treatment response [217–224]. We are currently conducting a randomized controlled trial comparing standard symptom severity-driven decision making for triaging and adapting level of care to the use of these multivariate predictive algorithms to drive decision making. Annual interim analyses are conducted to refine and update the algorithms so that by the end of the study, we will have an algorithm that includes the combination of variables most predictive of outcomes in order to triage and adapt care optimally. We thus hypothesize that the data-driven algorithm-based decision making will outperform the standard symptom severity-driven decision making, resulting in greater improvement in depression and anxiety. Findings from this trial will inform potential optimization of STAND and will advance intervention effectiveness by providing a tool to providers that can aid clinical decision-making to optimize both clinical outcomes and allocation of resources. Indeed, giving patients the level of care they need when they need it, and making rapid adjustments to that care is likely to be a cost-effective solution; cost-effectiveness will be evaluated in the final year of the five-year study.

In addition to these variables, we are beginning to explore behaviors and physiology in daily life, recorded via smart phones and apple watches, as predictors of treatment response. Features include sleep patterns, activity levels, social interaction and voice features, which may provide sensitive markers of both current need and tipping points of future needs [225, 226]. Passive monitoring may not only be a more sensitive index but may be the only index for participants who fail to complete the self-report symptom scales on a regular basis. However, digital sensing of anxiety and depression awaits validation and consistent replication before being incorporated into clinical decision-making.

Within the clinical care arm, decisions about which CBT strategy to select or whether to add pharmacotherapy are based on patient symptom reporting and clinical judgment. Predictive modeling to more rapidly and accurately identify who is likely to respond to a specific CBT intervention, or to pharmacotherapy, may prove more effective. Machine learning models already have been used to identify patients who respond differently to packaged psychological treatments such as CBT versus interpersonal psychotherapy [227], CBT versus eye movement desensitization and reprocessing [228], prolonged exposure versus cognitive processing therapy [229], and CBT versus psychodynamic therapy [230], and CBT versus person centered counseling [231]. Moreover, such modeling has proven effective for decision making regarding psychotherapy versus pharmacotherapy for depression [232]. Application of such modeling within the STAND clinical care arm will be a focus of future research. Another approach we will explore is mechanistically driven, and will build upon our current modular approach to therapeutic strategies. Currently, symptoms or problem areas (e.g., social anxiety) are matched to therapeutic strategies designed to target theorized mechanisms (e.g., cognitive misappraisal, avoidance behavior, poor extinction learning, rumination). In the future, with advances in cognitive, neural, and behavioral sciences, we may be better able to link symptom profiles to underlying mechanisms (e.g., deficits in extinction learning, hypo-responsiveness to reward anticipation, attentional bias to threat, over general memory, and stress reactivity), and identify which therapeutic strategies most effectively shift specific mechanisms [233, 234]. With such information, we can then assign those strategies that are most targeted at the mechanisms most relevant to a given symptom profile.

Other areas of optimization include aspects of coach training and supervision, which pose limits upon scalability. Digitization of coach training, as we have already implemented for mental health professionals [235], is already underway. Supervision itself can be made more scalable by having advanced coaches participate in a peer-review group since peer-led supervision is acceptable for lay therapists [236, 237].

## Challenges

Despite the increase in available treatments for anxiety and depression, the prevalence of these problems has not significantly reduced. In adolescents and young adults in particular, the prevalence of these problems is increasing according to epidemiological studies (see [70] for a review). This is partly due to lack of highly effective, scalable, targeted prevention interventions for children and adolescents [70]. Targeting risk factors before the onset of anxiety and depressive disorders holds the promise of reducing the burden of anxiety and depression in young adulthood and beyond, but these have been difficult to deploy and most prevention programs fail to target the factors associated with greatest risk of disorder onset [238]. To date, STAND has been implemented for individuals aged 18 years and older. A future challenge will be tailoring STAND for youth, not only for treatment but for prevention of anxiety and depression, in ways that target personally relevant risk factors. This tailoring is likely to include adaptations to all materials for developmental appropriateness, partnering with high school systems to implement STAND, and exploring the extent to which families will be involved in the students’ care, including addressing issues of confidentiality and stigma.

A second challenge pertains to uptake. Despite the promise of greater reach and scalability with the provision of digital mental health tools, the problem of uptake and retention in digital therapies is ubiquitous [239, 240] and is a challenge we continue to address in STAND. Despite wide-scale messaging, rates of students who complete STAND screening for depression and anxiety remains relatively low, consistent with rates for other studies of college mental health [241]. Tying screening to course registration may prove to be an effective strategy in the future. Once screened, attrition occurs across registration, assessment, and enrollment in a tier of care. We are currently exploring ways to increase STAND uptake, including text messaging, video testimonials [242], and fotonovelas [243] to reduce stigma and increase outcome expectancies. Once enrolled, peer coaching is designed to increase engagement and retention in digital CBT [180]. We are currently evaluating the value of matching participants with coaches on demographic characteristics.

Finally, evidence-based systems of care are difficult to sustain in real-world clinical settings and health care systems once research funding for these programs ends. There are many possible implementation factors responsible for this research-to-practice gap at the administrative and systems levels, many of which relate to funding streams. Working with clinic partners and health care systems early on to identify barriers and facilitators to adopting and sustaining these interventions in clinical settings is critical for ensuring that effective tools make their way to individuals. For example, we continue to work closely with our Los Angeles County DMH and California Community College stakeholders. In addition to meeting regularly with community college administrators and DMH leadership to address implementation barriers in real time, we gather detailed annual feedback from a diverse group of stakeholders and make refinements to STAND based on that feedback. We also host annual stakeholder retreats to gather input about key challenges in implementing and sustaining STAND. For future sustainability, we are in discussion with community college leaders to identify pathways for funding. We also plan to develop an online tool for community college administrators to request the elements of STAND they would like to implement on their campuses that will provide budgetary information they can use to request funds from the state. Sustainability and large-scale roll-outs of effective programs for young adult mental health such as STAND is a complex challenge that relies on close partnerships, interdisciplinary collaboration, and creativity.

## Footnotes

1 Other versions of STAND are available for specific populations, such as perinatal depression [187].
